# Morphology of the acromioclavicular-joint score (MAC)

**DOI:** 10.1007/s00402-022-04407-3

**Published:** 2022-04-05

**Authors:** Milad Farkhondeh Fal, Marius Junker, Konrad Mader, Karl Heinz Frosch, Jörn Kircher

**Affiliations:** 1 Department of Trauma and Orthopaedic Surgery, University Hospital Hamburg Eppendorf, Martinistraße 52, 20251 Hamburg, Germany; 2 Department of Shoulder and Elbow Surgery, ATOS Klinik Fleetinsel Hamburg, Admiralitätstrasse 3-4, 20459 Hamburg, Germany; 3 Medical Faculty, Heinrich-Heine-University Düsseldorf, Moorenstr. 5, 40255 Düsseldorf, Germany; 4 Orthopaedic University Hospital Friedrichsheim, Marienburgerstr. 2, 60528 Frankfurt, Germany

**Keywords:** Acromioclavicular joint, Score, Degenerative joint disease, Osteoarthritis, Shoulder, Morphology, MRI

## Abstract

**Introduction:**

To date there is no generally accepted specific definition or classification of acromioclavicular (AC) joint osteoarthritis. The aim of this study is to analyze morphological parameters using magnetic resonance imaging (MRI) and to develop a scoring system as a basis for decision making to perform an AC-joint resection.

**Materials and methods:**

In a retrospective-monocentric matched pair study, healthy and affected subjects were investigated using T2 MRI scans in the transverse plane. There were two groups, group 1 (*n* = 151) included healthy asymptomatic adults with no history of trauma. In group 2, we included *n* = 99 patients with symptomatic AC joints, who underwent arthroscopic AC-joint resection. The central and posterior joint space width and the AC angle were measured. Morphological changes such as cartilage degeneration, cysts and bone edema were noted. Malalignment of the joint was defined as: posterior joint space width < 2 mm in conjunction with an AC angle > 12°. A scoring system consisting of the measured morphologic factors was developed.

**Results:**

Symptomatic and asymptomatic patients showed significant differences in all measured items. We observed a significant difference in the MAC score for symptomatic and asymptomatic patients (mean 10.4 vs. 20.6, *p* = 0.0001). The ROC (receiver operator characteristic) analysis showed an excellent AUC of 0.899 (*p* = 0.001). The sensitivity of the MAC score was 0.81 and the specificity 0.86. The MAC score shows a significant moderate correlation with age (*r* = 0.358; *p* = 0.001). The correlation of age and the development of symptoms was only weak (*r* = 0.22, *p* = 0.001). Symptomatic patients showed significantly more frequent malalignment compared to asymptomatic patients (*p* = 0.001), but the positive predictive value that a patient with malalignment is also symptomatic is only 55%.

**Conclusion:**

Patients with symptomatic AC joints showed a typical pattern of morphological changes on axial MRI scans with early posterior contact of the joint surfaces, reduction of joint space and malalignment as the basis for the development of a scoring system. The MAC score shows excellent test characteristics, and therefore, proved to be both an appropriate guidance for clinical practice as well as an excellent tool for comparative studies and is superior to the assessment of malalignment alone.

**Level of evidence:**

Level IV, retrospective diagnostic study.

## Introduction

Shoulder function is based on an intact complex anatomical structure of the glenohumeral joint, the acromio-clavicular (AC) joint, sterno-clavicular (SC) joint and the scapulo-thoracic joint and the adjacent tissues. Clinical symptoms due to structural changes of one or more parts usually present as a challenge to diagnostics and treatment decisions due to the similarity and variability of symptoms [15, 33]. The AC joint is subject to degenerative changes in terms of overload and or traumatic events during fall on the arm and shoulder, inflammatory arthritis and others and is getting more attention as a source of shoulder pain in recent [8, 15, 16, 19, 24]. In the early stages of the disease, most of the patients are asymptomatic even with apparent radiological changes and sometimes even advanced joint destruction [12, 29]. The primary therapeutic approach is conservative treatment with physiotherapy, anti-inflammatory and narcotic pain medication, intra-articular injection with corticosteroids and local anesthetics [11, 27, 32].

Usually failed conservative therapy over a time period of months is the indication for AC-joint resection or resection of the lateral clavicle [14]. This procedure can be performed either in an open fashion or arthroscopically [21, 28, 31]. Because of the preservation of the superior capsule, the preference and better outcome are attributed to the arthroscopic technique nowadays [4, 7, 15, 23, 26]. Recently, an increased incidence of arthroscopic AC-joint resection in the general population can be observed, although clear clinical guidelines in terms of the right timepoint and amount of structural changes, that can be tolerated are missing [2, 20].

Little is known about the pathophysiology of non-traumatic degenerative AC-joint disease. In our clinical practice, we made the observation that the morphological changes seem to follow a typical pattern beginning at the posterior part with a certain amount of malalignment.

The aim of this study is (1) to analyze morphological parameters of AC-joint degeneration on axial MRI scans and (2) to develop a scoring system that can help to find the right indication and timepoint for AC-joint resection.

## Materials and methods

This is a retrospective-monocentric matched pair diagnostic study in which healthy and affected subjects were investigated chronologically using MRI scans in the transverse plane. All patients signed informed consent before participation and the ethics committee approved the study (study number: 3429).

Included in group 1 were healthy subjects with normal asymptomatic AC joints and in group 2, patients with symptomatic AC joints who underwent arthroscopic AC-joint resection.

Exclusion criteria were all relevant pathologies or activities that affect the AC joint such as history of trauma, previous surgery and participation in overhead athletic sports.

**Fig. 1:**
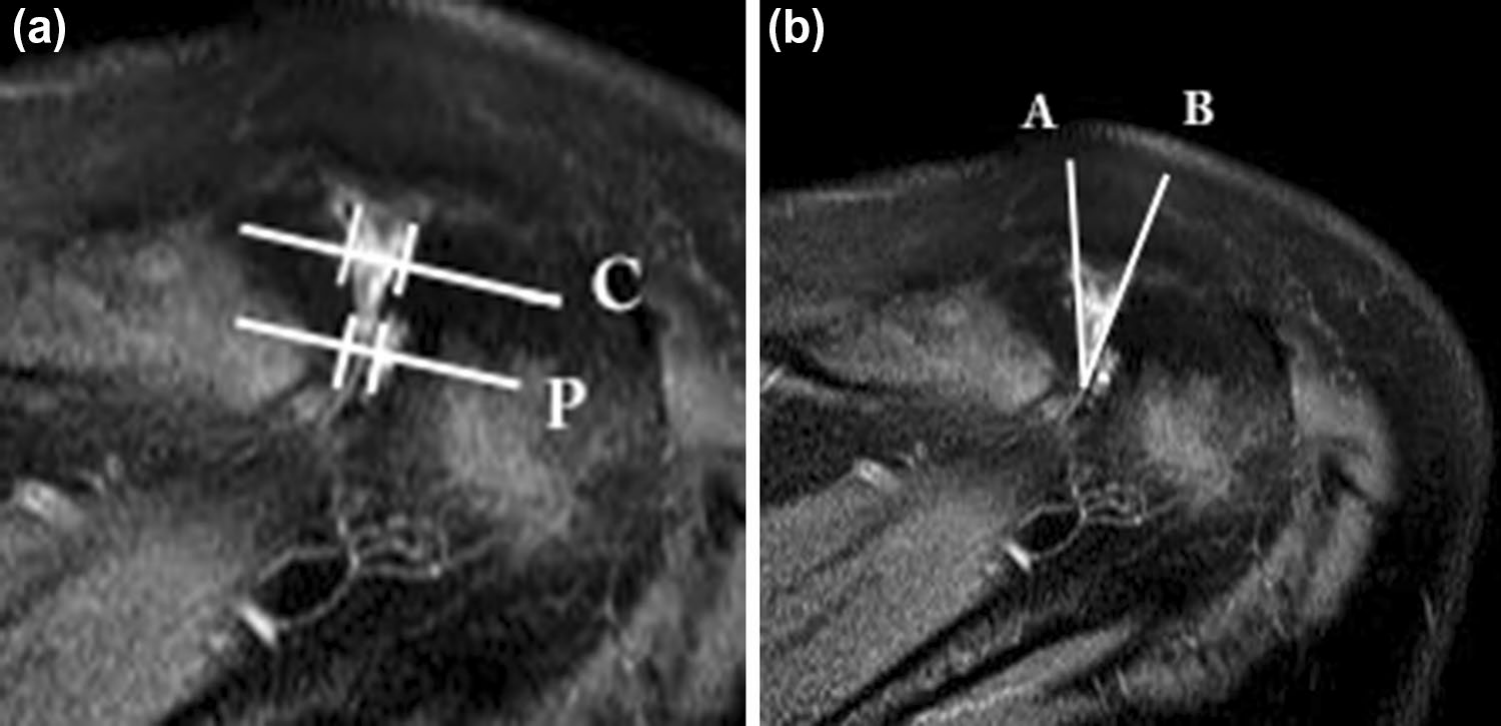
**a** On MRI scans in the transverse plane with fluid-sensitive sequences (T2-weighted images, T1-weighted images with contrast medium or comparable sequences), the central and posterior joint space width of the AC joints was measured. The distance between both perpendicular bars on line C represents the central joint space width and the distance between both perpendicular bars on line P represents the posterior joint space width. **b** The AC angle was measured between two lines that are aligned with the joint surface of the acromion (line A) and the lateral clavicle (line B), respectively (an anteriorly open angle defined with positive values)

On MRI scans in the transverse plane with fluid-sensitive sequences (T2-weighted images, T1-weighted images with contrast medium or comparable sequences), the central and posterior joint space width of the AC joints was measured (Fig. 1A). The AC angle was measured between two lines that are aligned with the joint surface of the acromion and the lateral clavicle, respectively (an anteriorly open angle defined with positive values) [10] (Fig. 1B). Morphological changes such as cysts, bone edema and cartilage degeneration were noted on both joint partners. Cartilage grading was performed based on the criteria of Petersson et al.: Grade I: superficial degenerative signs with blister formation and some fragmentation; Grade II: deep degeneration with cartilage fragmentation, blister formation and penetrating ulceration of the joint surface in irregularly shaped areas; Grade III: full-thickness cartilage degeneration denuding the subchondral bone of more than 50% of the joint surface [24].

Malalignment of the AC joint was defined as a posterior joint space < 2 mm and an AC angle < 12° [10].

All measurements were independently performed by the first and second authors, in a blinded fashion, on a computer using specific PACS software (dicomPACS View, Version 5.2.11, Oehm and Rehbein, Rostock, Germany). Average values were used for further statistical analysis such as group comparison. The raters were instructed to measure and note the above-mentioned measurements and changes in the first plane where both articular surfaces were adequately seen, starting distally.

SPSS^®^ software (Version 27.0; IBM Corp., Armonk, NY, USA) was used for statistical analysis. Measurement values were reported as mean ± standard deviation (SD) and range with 95% confidence intervals. The normality of data was tested by visual inspection using boxplots and scatterplots and statistically using the Kolmogorov–Smirnov and Shapiro–Wilk tests. As the normality assumption was uncertain in portions of the data, the *U*-test according to Mann–Whitney and the Wilcoxon rank test were used to compare means of both groups and categorial data. The Spearman’s rank test was used to perform correlation analysis.

The MAC test characteristics were analyzed using the ROC (receiver operator characteristic) curve.

Inter-observer reproducibility of the measurements was quantified with the intraclass correlation coefficient (ICC) using a pair-wise correlation model with absolute agreement. The ICC was classified from fair to excellent [5, 13]. *p* values below 0.05 were considered to be statistically significant.

## Results

Symptomatic and asymptomatic patients (group 1 and group 2) showed significantly different values for each item of the MAC score (Table 1).

**Table 1:** Measured and assessed items of the MAC score and the MAC score for group 1 and group 2

	Group 1				Group 2				
	Mean	Min	Max	SD	Mean	Min	Max	SD	p-value
AC angle	14.9	-4	41.3	9.5	11.9	-10.6	43.2	9.0	0.024
Joint space width central	2.1	0.0	5.6	1.3	3.0	0.0	7.3	1.5	0.001
Joint space width posterior	1.4	0.0	7.3	1.7	3.4	0.0	10.3	2.1	0.001
Cartilage grading acromion	2.9	2.0	3.0	0.3	2.2	1.0	3.0	0.8	0.001
Cartilage grading clavicle	2.9	1.0	3.0	0.3	2.2	1.0	3.0	0.8	0.001
Bone marrow edema acromion	0.7	0.0	1.0	0.4	0.5	0.0	1.0	0.4	0.001
Bone marrow edema clavicle	0.8	0.0	1.0	0.4	0.5	0.0	1.0	0.4	0.001
MAC score	10.4	2	22	5.2	20.6	9	30	5.6	0.001

AC angle in degree, joint space width in millimeter, cartilage and bone marrow grading and MAC score in absolute numbers. Group comparison using the Mann–Whitney test (p-value < 0.05 significant, 95% confidence interval)

Gender distribution was equal for both groups but the symptomatic patients were significantly older (Table 2).

**Table 2:** Age distribution for groups and gender in absolute values in years. *P* value for comparison between genders in the groups according to Mann–Whitney

	Mean	Min	Max	SD	p-value
Group 1					
All (n = 151)	50.2	15.1	81.5	18.7	
Female (n = 65)	51.2	16.0	65.5	19.6	
Male (n = 86)	49.5	15.1	79.4	18.2	0.628
Group 2					
All (n = 99)	60.1	18.2	84.5	10.6	
Female (n = 47)	60.2	28.9	77.3	10.9	
Male (n = 53)	60.0	18.2	84.5	10.6	0.898

The interobserver reliability ranged from excellent to good (Table 3).

**Table 3:** Reliability analysis using the intraclass-correlation-coefficient (ICC) (two-way mixed, single measures, consistency, 95% confidence interval)

	ICC	p-value
AC angle	0.970	0.001
Joint space width central	0.692	0.001
Joint space width posterior	0.898	0.001
Cartilage grading acromion	0.898	0.001
Cartilage grading clavicle	0.903	0.001
Bone marrow edema acromion	0.811	0.001
Bone marrow edema clavicle	0.817	0.001

**Fig. 2:**
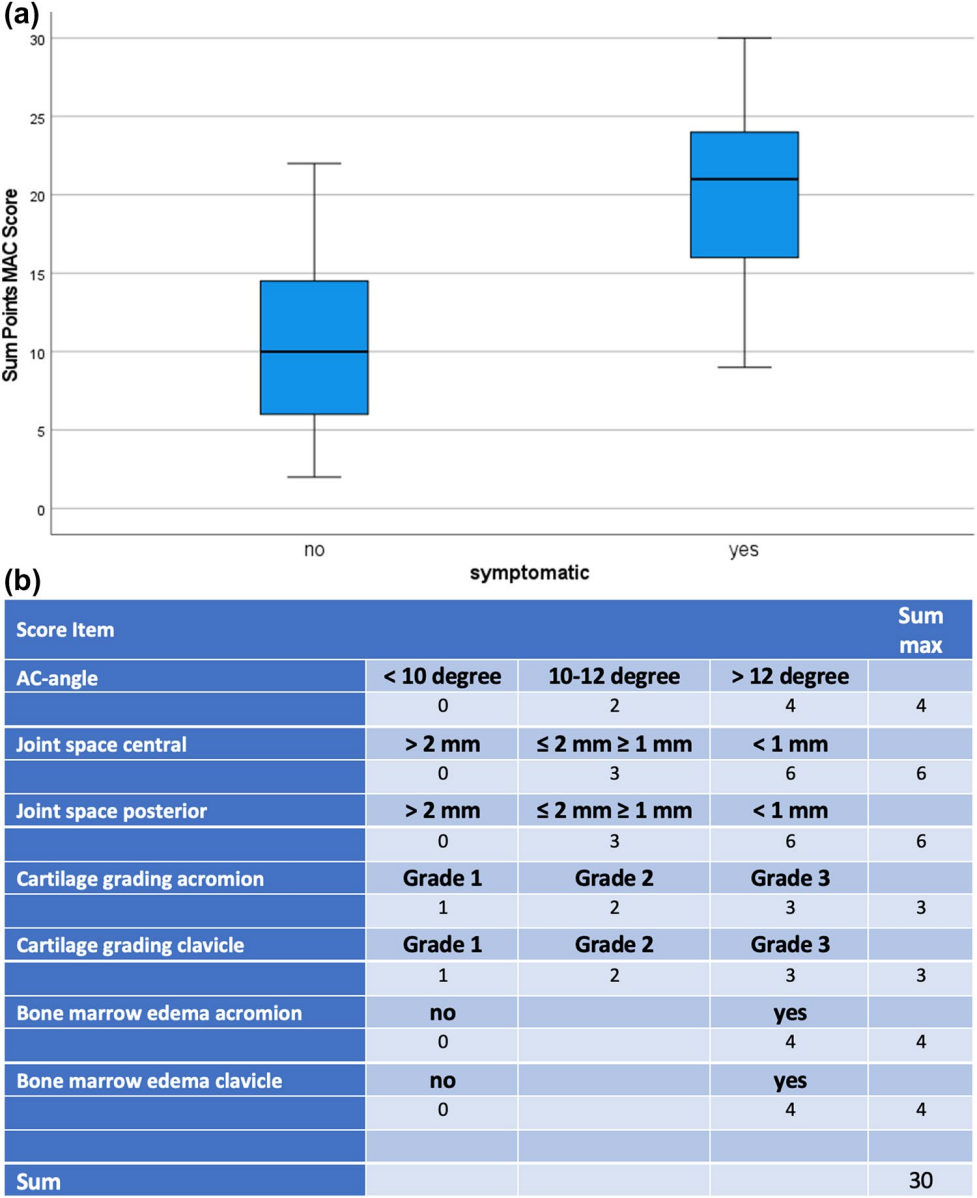
**a** Significant difference in the MAC score for asymptomatic (left) and symptomatic (right) patients. **b** MAC score: seven items (left column) are assessed and given points according to the defined criteria (column 2–4). The right column lists the maximum points achievable, the highest possible score is 30 points. AC angle: the angle between a line parallel to the joint surface of the acromion and the lateral clavicle, anteriorly open angles are positive (Fig. 2). Joint space central: the joint space width at the center of the AC joint in the anterior–posterior direction (Fig. 1). Joint space posterior: the joint space width at the most posterior part of the AC joint in the anterior–posterior direction (Fig. 1). Cartilage grading acromion and clavicle: the hyaline cartilage at the acromial part or the clavicle part of the AC joint, respectively, is graded according to Petersson et al. Grade I: superficial degenerative signs with blister formation and some fragmentation; Grade II: deep degeneration with cartilage fragmentation, blister formation and penetrating ulceration of the joint surface in irregularly shaped areas; Grade III: full-thickness cartilage degeneration denuding the subchondral bone of more than 50% of the joint surface. Bone marrow edema acromion and clavicle: the presence of a bone marrow edema at the acromial part or the clavicle part of the AC joint, respectively, is assessed

We observed a significant difference in the MAC score for symptomatic and asymptomatic patients (mean 10.4 vs. 20.6, *p* = 0.0001) (Table 1) (Fig. 2A).

**Fig. 3:**
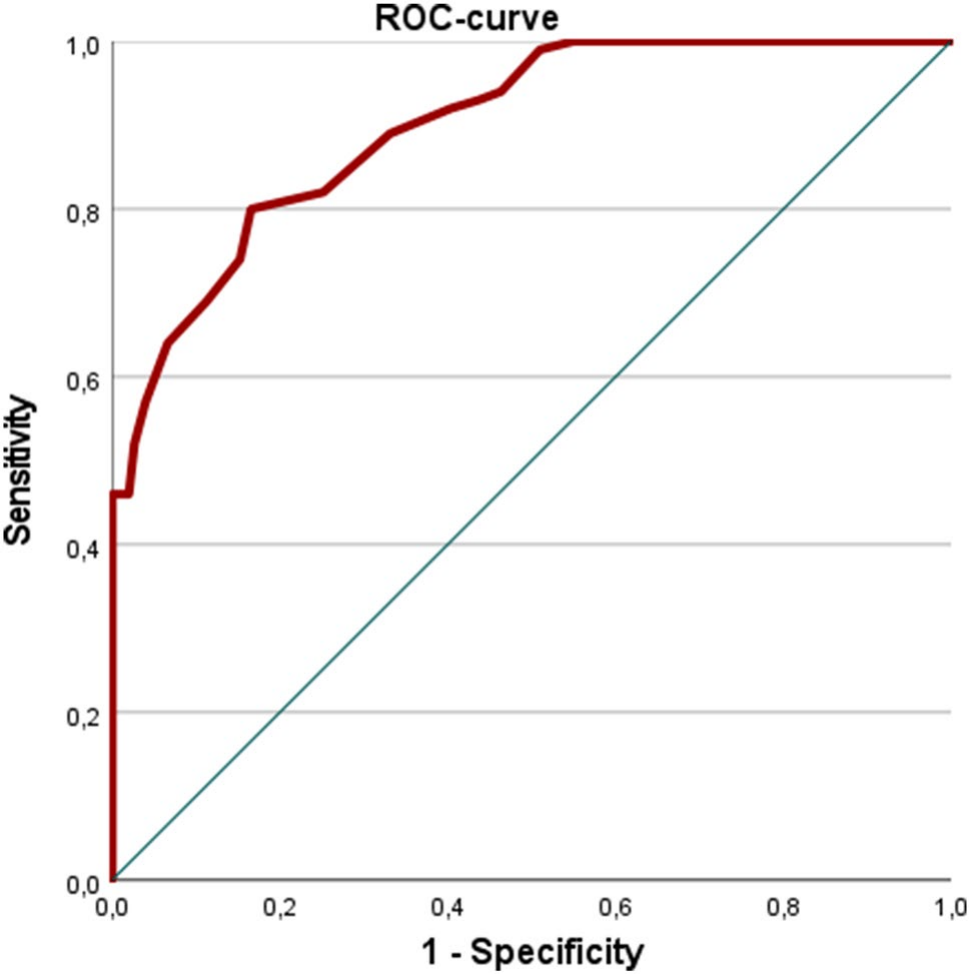
ROC-curve (receiver operator characteristics curve) for the MAC score. The area under the curve (AUC) is 0.899, the standard error 0.019

The ROC (receiver operator characteristic) analysis showed an excellent AUC of 0.899 (*p* = 0.001) (Fig. 3). With a cutoff at 15 points, the sensitivity of the MAC score was 0.81 and the specificity 0.86. The MAC score shows a significant moderate correlation with age (*r* = 0.358; *p* = 0.001). The correlation of age and the development of symptoms was only weak (*r* = 0.22, *p* = 0.001).

Symptomatic patients showed significantly more frequent malalignment compared to asymptomatic patients (p = 0.001), but the positive predictive value that a patient with malalignment is also symptomatic is only 55%.

## Conclusions

The most important finding is the fact that symptomatic patients showed a typical pattern of malalignment and morphological changes of the AC joint in the axial plane beginning with posterior joint space narrowing, subsequent opening of the AC angle and degenerative changes of the cartilage both on the acromion and the clavicle with subsequent development of bone marrow edema in both joint partners. Painful symptoms are attributed to patients showing these radiological features.

It has been shown that advanced degenerative changes can be well visualized on ap radiographs but it cannot depict smaller changes [30]. Actually, we could show that in a number of cases the affected AC joints show a tendency to open up the anterior joint space and the AC angle anteriorly which could easily be mistaken for as a normal joint space on ap radiographs.

In a radiological study by de Abreu et al. [6], morphological finding compatible with the degenerative AC joint were much more frequently detected with MR imaging than with radiography. In addition, severity of the disease was judged more severe with MRI. Especially, the visualization of cysts within the acromion and clavicle was significantly more frequent and sensitive with MR imaging.

Edelson et al. described a typical pattern of changes at the acromion in advanced degenerative changes of the AC joint with elongation of the joint facet in the sagittal plane principally in the posterior aspect of the acromial facet [8]. This is confirmed by our results that the posterior contact of the distal clavicle with the acromial joint facet is a typical phenomenon in the affected patient group. It appears to be logical that by time and continued overload with formation of osteophytes in conjunction with the deformation of the bony surface flattening of the joint surface and extension in the posterior part occurs.

We are not aware of any other study that assessed the morphologic changes of degenerative AC-joint disease in the axial plane and that described the joint space width in asymptomatic adult subjects [29].

Recently, Bomfim et al. [3] showed in their cross-sectional study that bone edema in the MRI and also in the histological examination were more frequent in patients having symptomatic AC joints but with only one radiologist as examiner and in a rather small group of subjects (*n* = 41).

This is in line with our results, where bone edema is a significant sign of the symptomatic degenerative AC joint and patients with subchondral bone edema in the magnetic resonance imaging were much more often found in the surgery group.

Petersson et al. assessed the AC-joint space in 151 patients on antero-posterior radiographs with a ruler on plain films [25]. They observed a decrease of the joint space with increasing age but did not describe a statistical test for it. This is confirmed by the results of our study, that shows a significant, but weak correlation of age and degenerative changes of the AC joint as expressed by the joint space width (Table 2). The fact, that the correlation is only weak underlines the possible explanation, that osteoarthritis in general and AC-joint degeneration in particular is believed to be the result of multifactorial influences of which age is only one important factor [1, 17, 18].

The normal acromio-clavicular joint space width is reported to be 1–3 mm [33] in a study of Zanca and 3.1 ± 0.8 by Petersson [25] in the frontal plane on anterior–posterior radiographs. It needs to be noted that many AC joints show a complex three-dimensional orientation (oblique in the frontal and axial plane) with individual variations and the radiograph is the sum of overlap of these structures [9]. Therefore, it becomes very likely, that a distinct measurement on axial MRI scans without overlap results in higher absolute values. The results of the study of Farkhondeh Fal et al. confirm this hypothesis with mean values for the anterior, central and posterior joint space width on axial MRI scans in a normal control group of 6 mm, 3 mm and 4 mm, respectively [10].

Nicholson et al. also found an increasing number of AC joints with degenerative changes with increasing age in an analysis of 420 scapulae, but in contrast to our study, the patient population consists of many elderly patients. In addition, the authors did not assess the joint space width but made a visual judgment based on the formation of osteophytes, erosion of the subchondral bone or the presence of eburnated bone [22].

Our study shows the importance to add advanced diagnostic imaging using MRI to the assessment of conventional radiographs. Despite the fact, that proper angulation and good image quality for axial radiographs is hard to achieve in clinical practice, the assessment of malalignment alone has an insufficient positive predictive value of only 55% for a patient to be symptomatic. The MAC score shows a robust and excellent test characteristic with an excellent AUC, very good sensitivity and specificity that makes it suitable for clinical practice in terms as an adjunct in decision making when to perform an AC-joint resection.

We are not aware of any other assessment tool that would allow the comparison of the degree of AC-joint degeneration in clinical studies. At this time we cannot make any comment, whether the degree of AC-joint degeneration as expressed by the MAC score is related to clinical outcome after AC-joint resection, but further studies will be able to show.

## Limitations

A selection bias is likely for patients in group 2 because the decision for arthroscopic AC-joint resection in this study was a combination of many factors: failure of conservative therapy, psychological strain, clinical examination, history of complaints and radiologic signs of joint degeneration and not only radiologic signs alone. All assessed items, especially the clinical examination, owns a substantial subjective bias in the person of the examiner.

The data were measured on MRI scans from different institutions and different scanners (1.5 T and 3 T). Although the scanning protocols are standardized some degree of variation must be assumed both for image quality and the definition of landmarks for the scanning by each radiographer and during the assessment of the examiner.

The MAC score has been established using retrospective data, therefore, prospective clinical studies using the MAC score are needed to validate the clinical impact.

With this study, we were able to identify typical morphological changes in patients with symptomatic AC joints, starting at the posterior part of the joint with joint space narrowing and subsequent degeneration of cartilage and the development of bone marrow edema which leads to an anatomic malalignment.

We were able to develop a scoring system (MAC score) that allows for a numerical assessment of the degree of degenerative changes (Fig. 2B). The MAC score shows excellent test characteristics and is suitable for both providing guidance in clinical practice to find the right indication and timepoint to perform AC-joint resection and as a tool to objectively compare AC-joint degeneration in future studies.
